# Supplementary material for: Endosomal mTORC2 Is Required for Phosphoinositide-Dependent AKT Activation in Platelet-Derived Growth Factor-Stimulated Glioma Cells
Source: Cancers (Basel). 2021 May 16;13(10):2405. doi: 10.3390/cancers13102405 (PMC8157044; doi:10.3390/cancers13102405)

Fig 1A

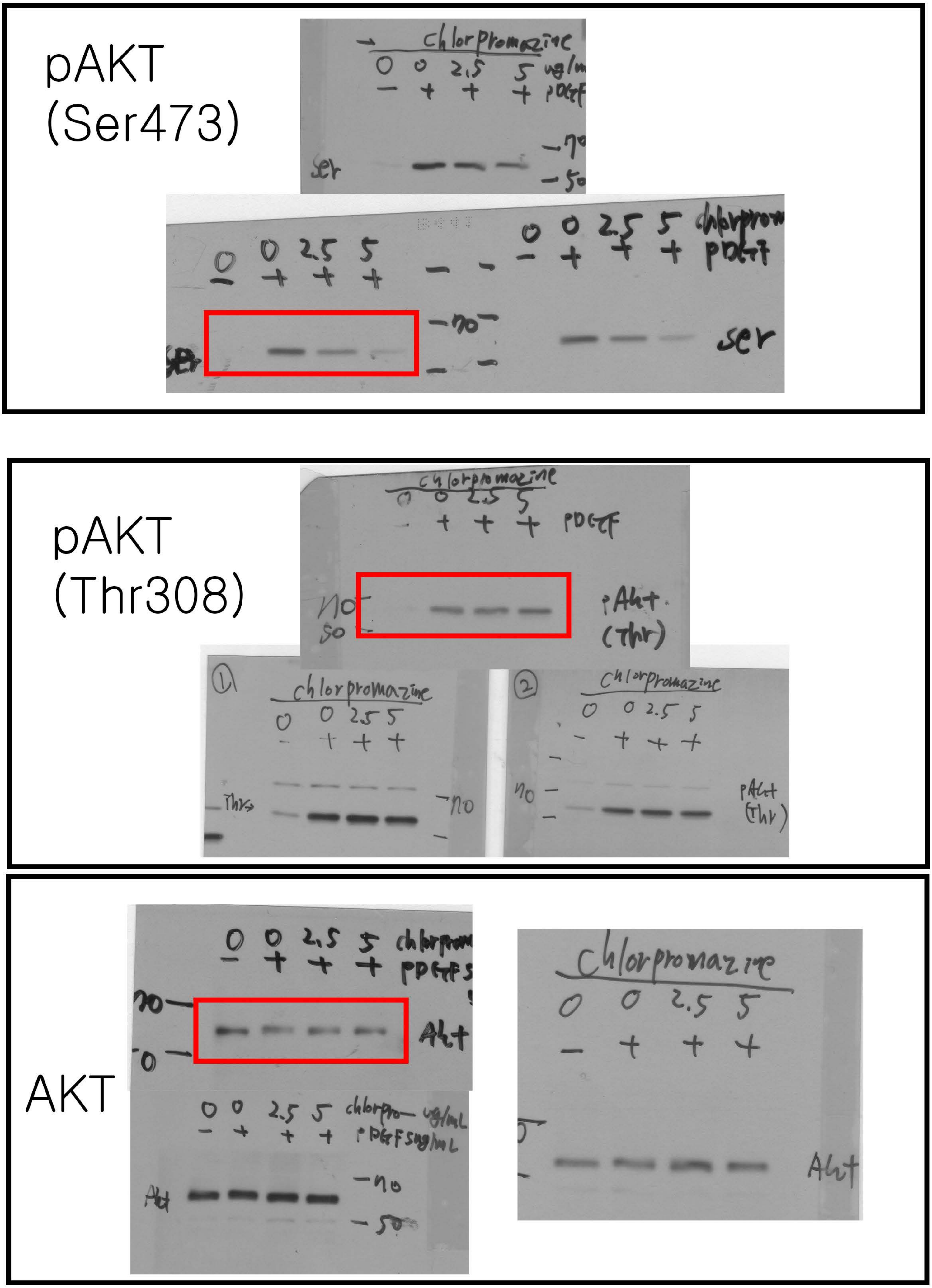

Fig 1C

pAKT (Ser473)

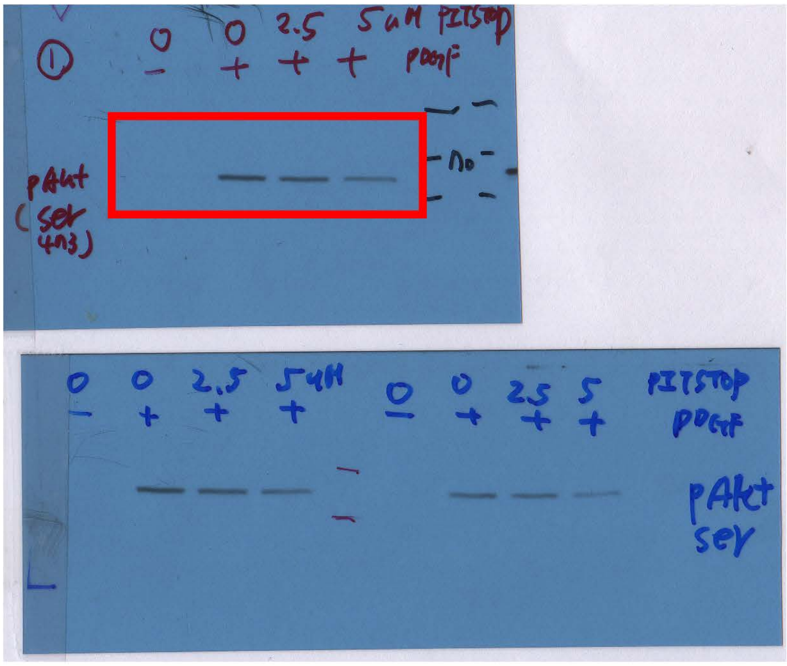

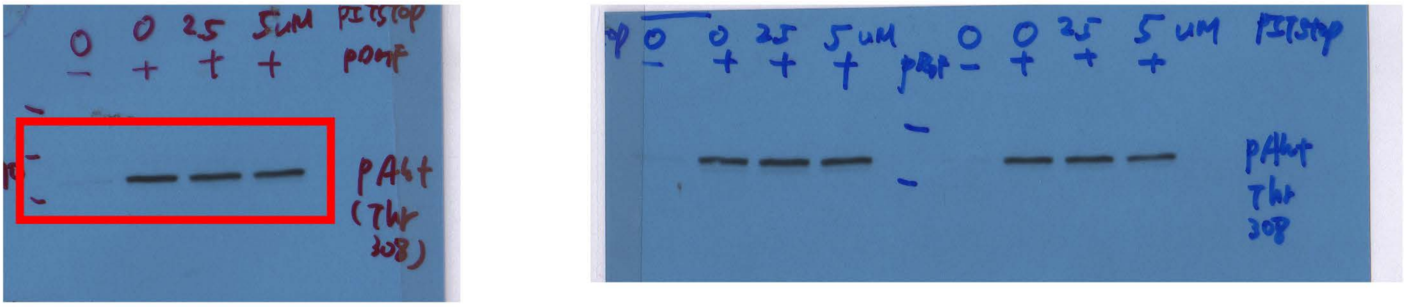

pAKT(Thr308)

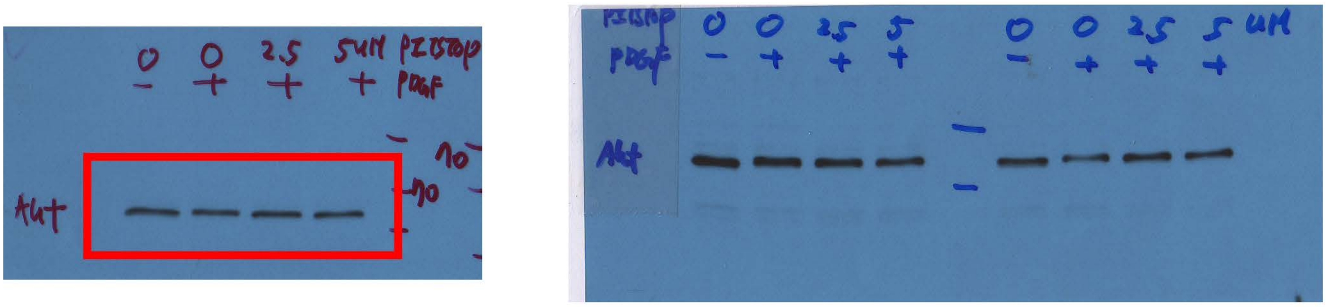

AKT

Fig 3B

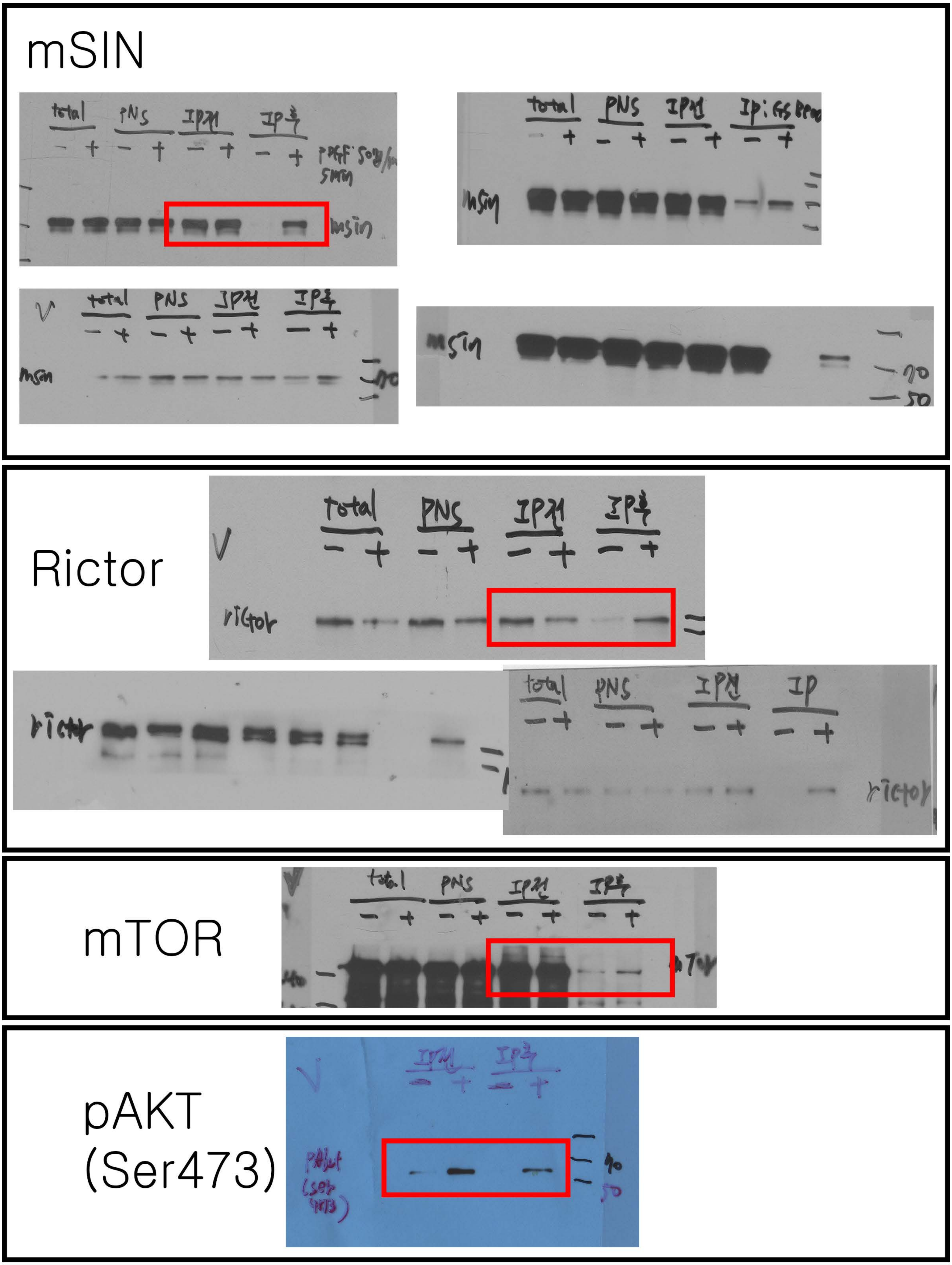

Fig 3B

Rab7

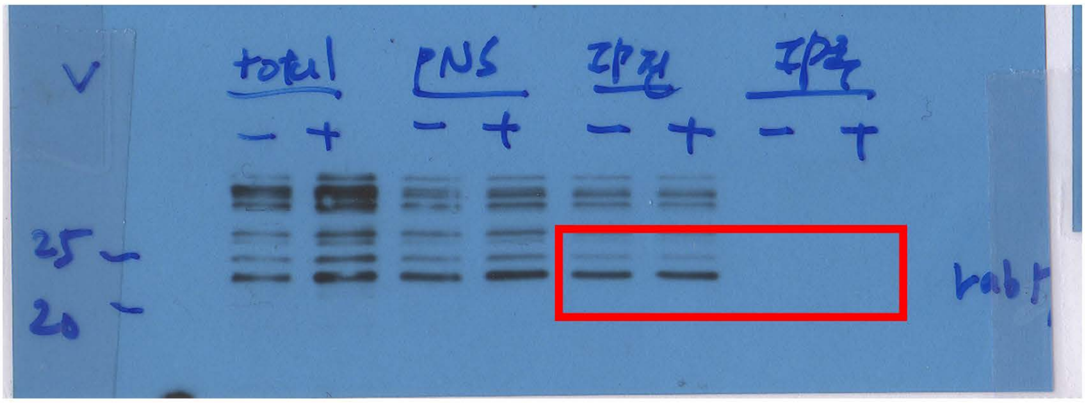

Cathepsin D

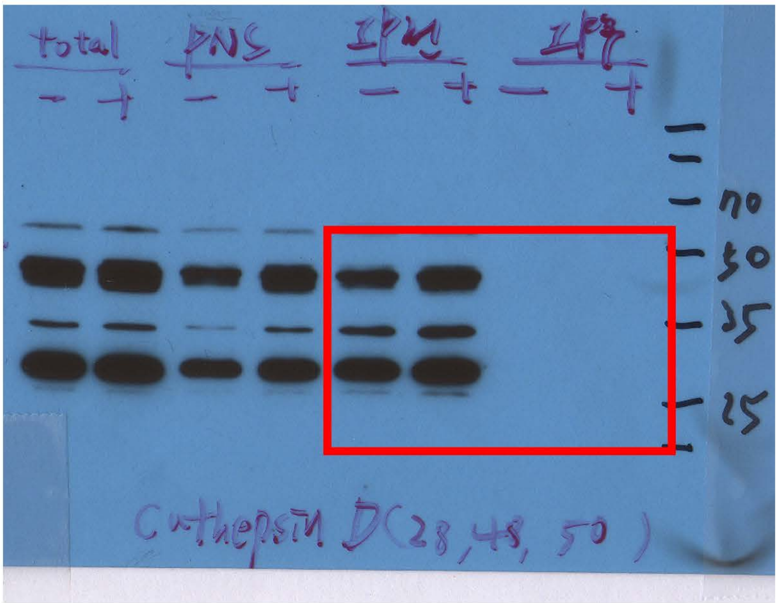

GST

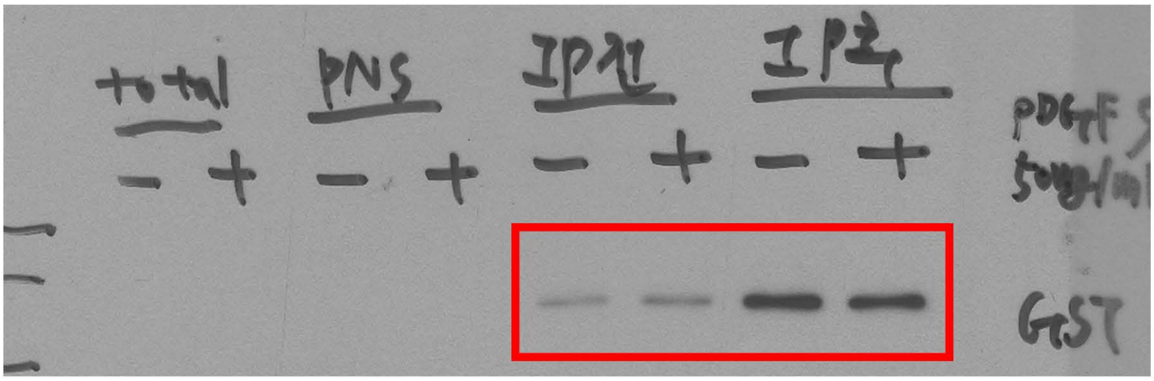

Fig 3B

Raptor

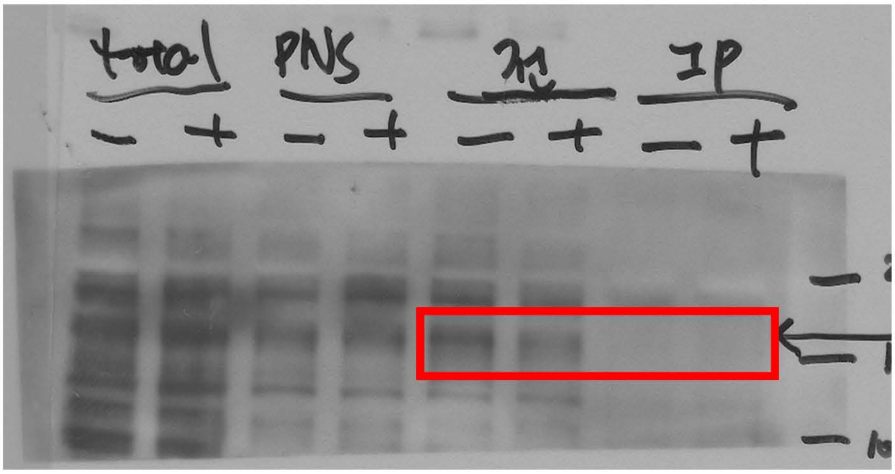

Rab5

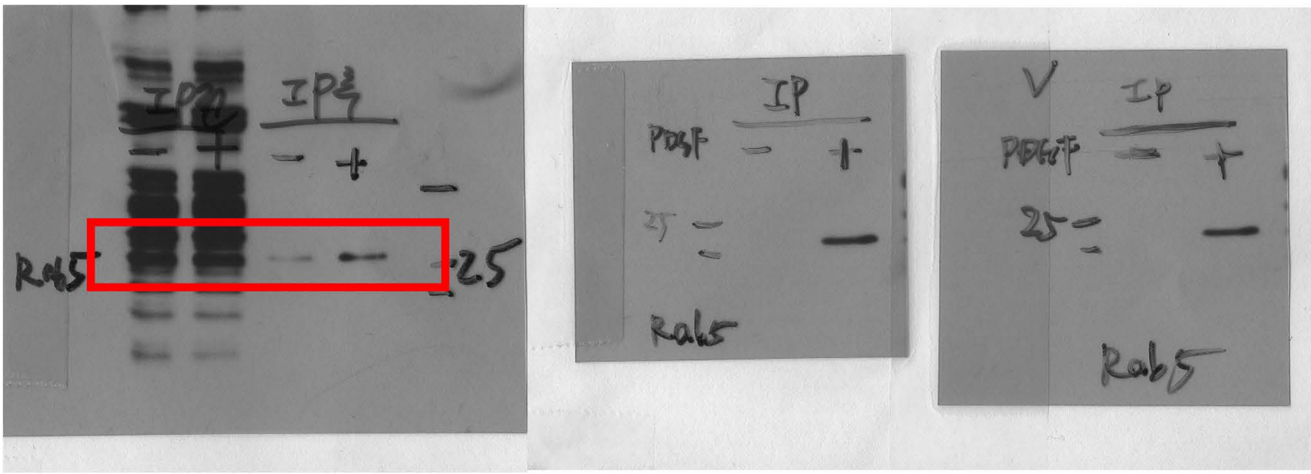

EEA1

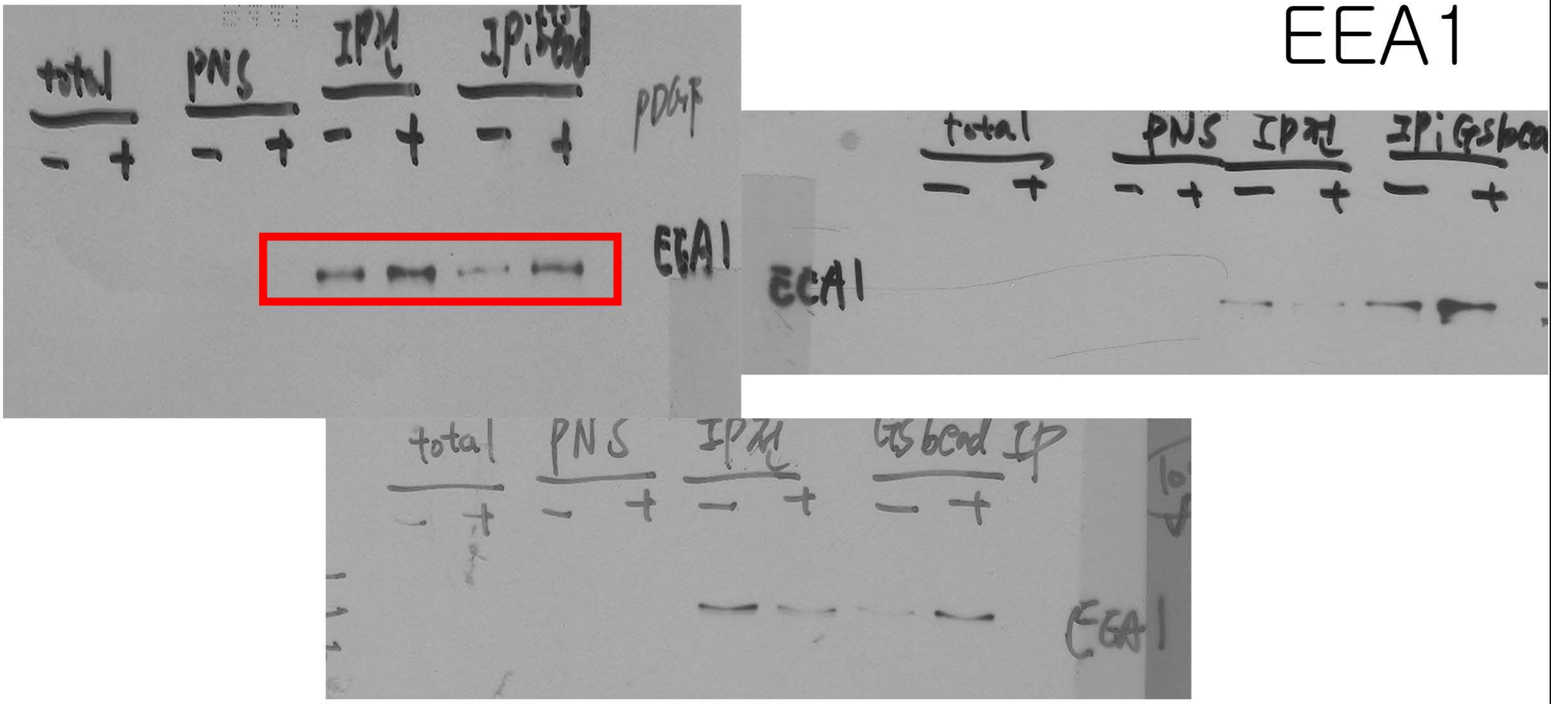

Fig 6A

AKT

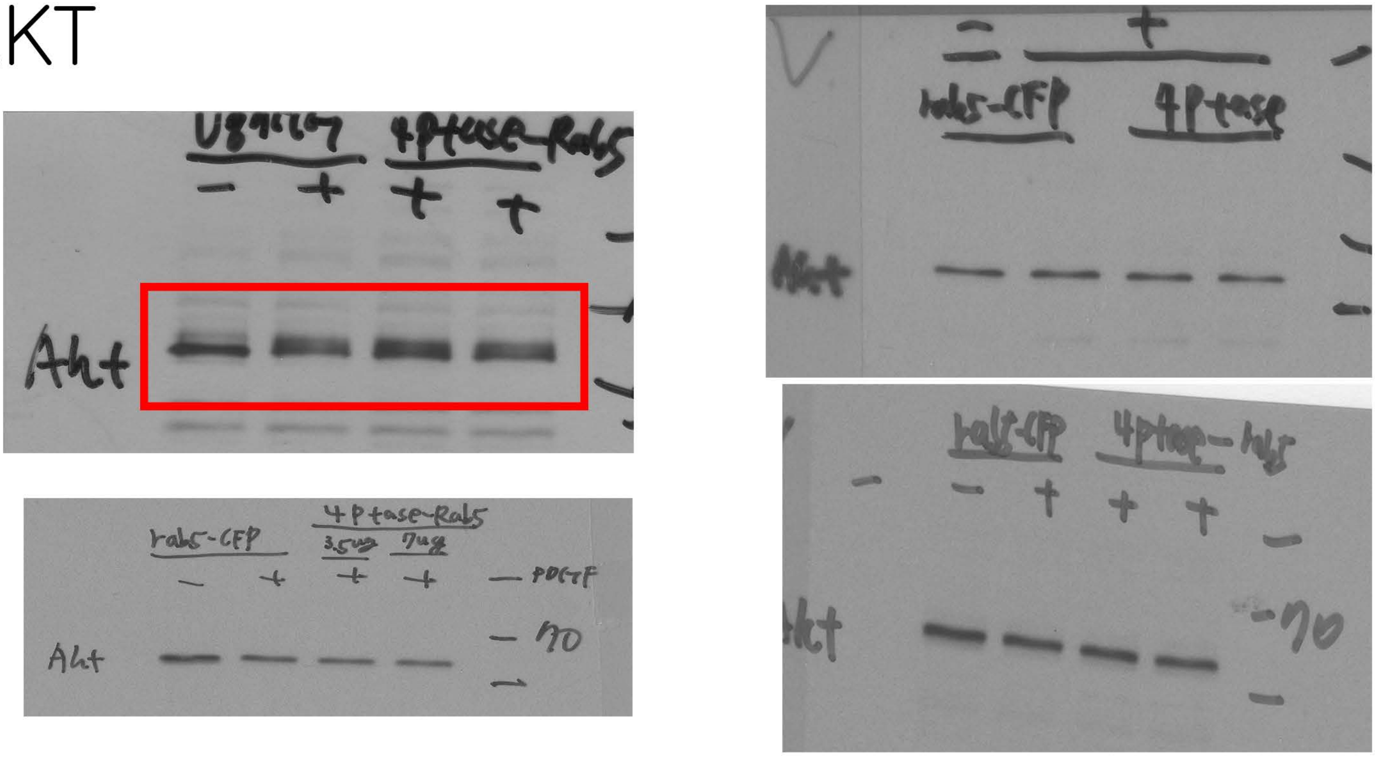

pFoxO1/3a

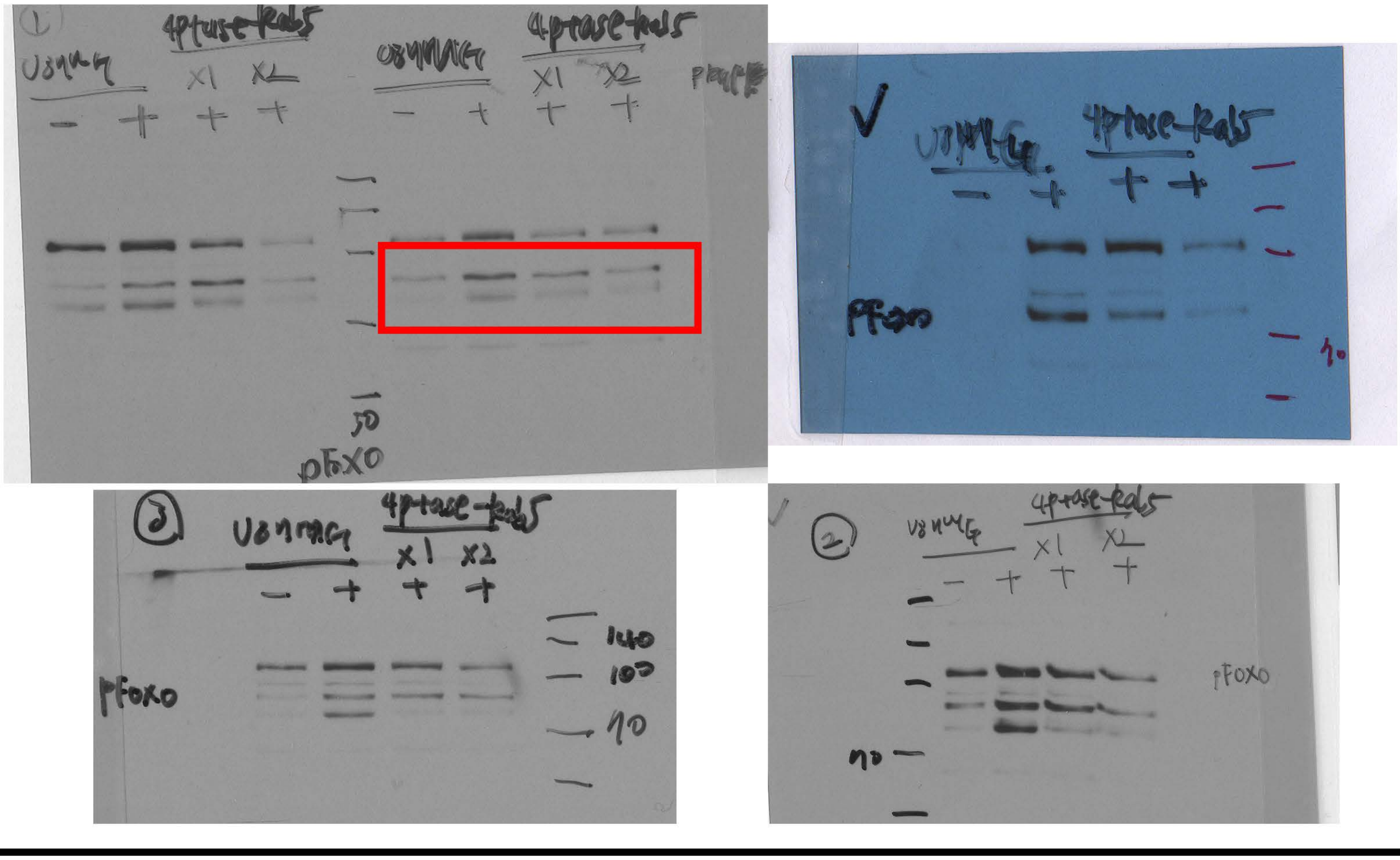

Fig 6A

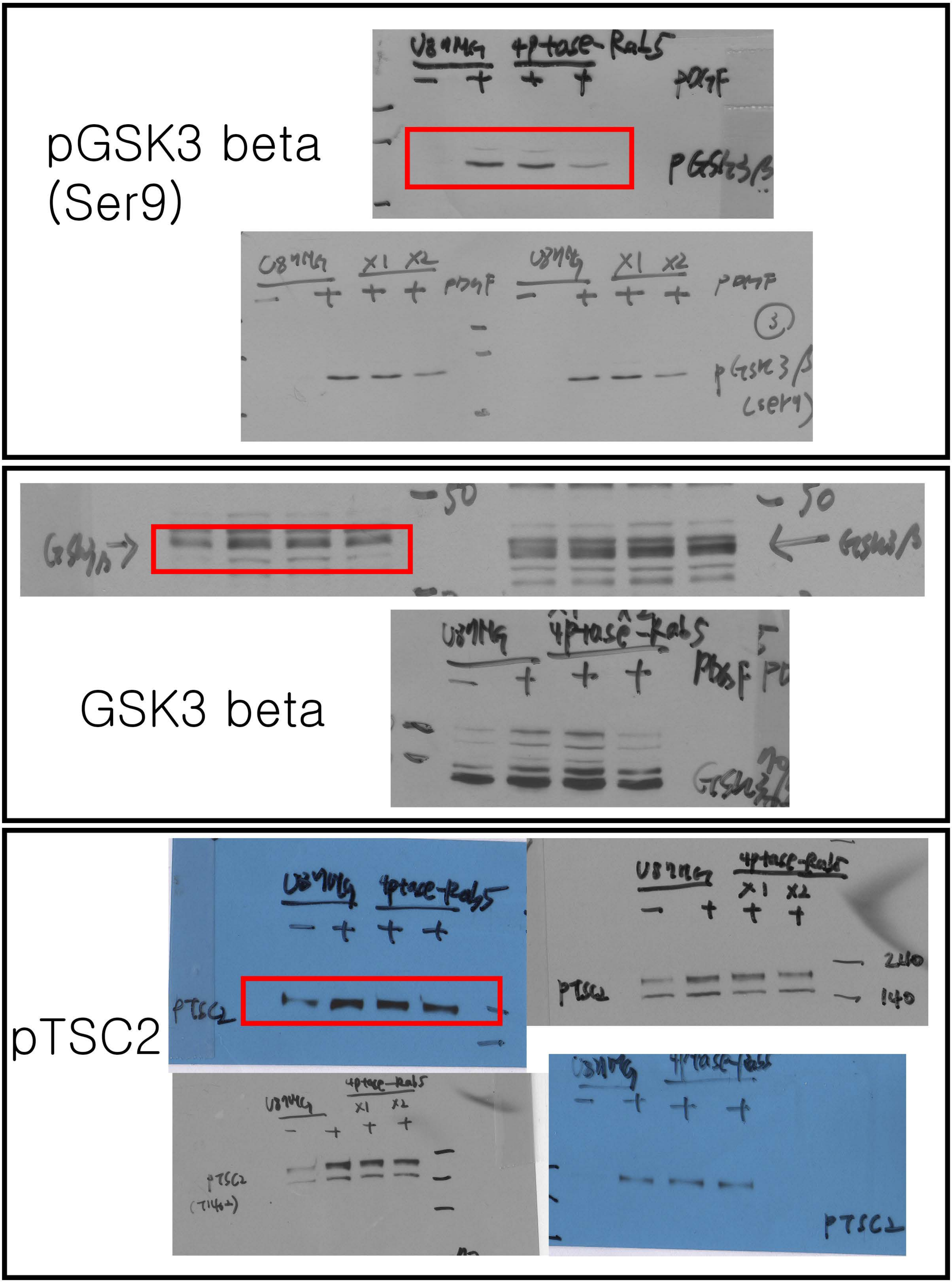

Fig 6A

pAKT(Ser473)

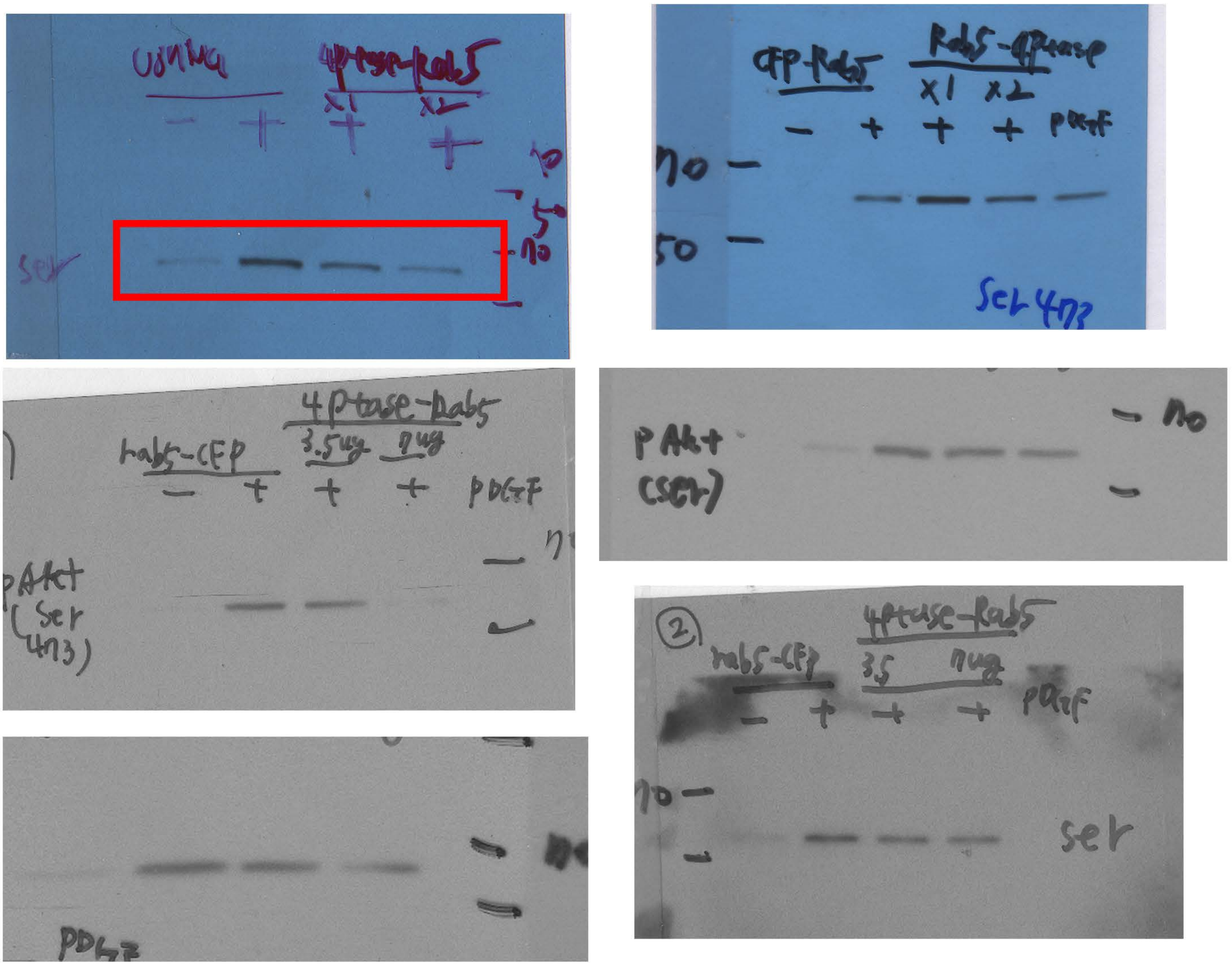

pAKT(Thr308)

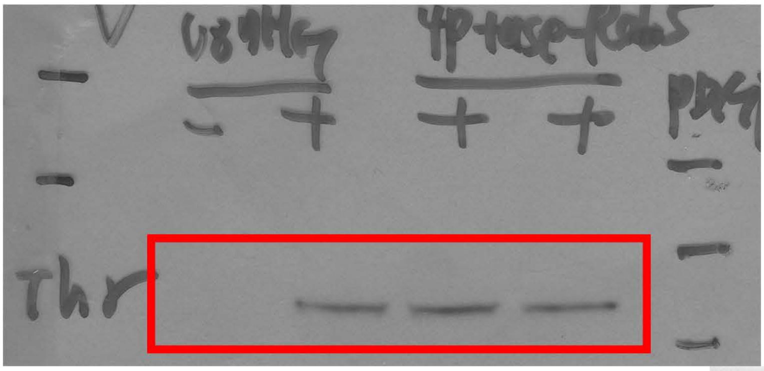

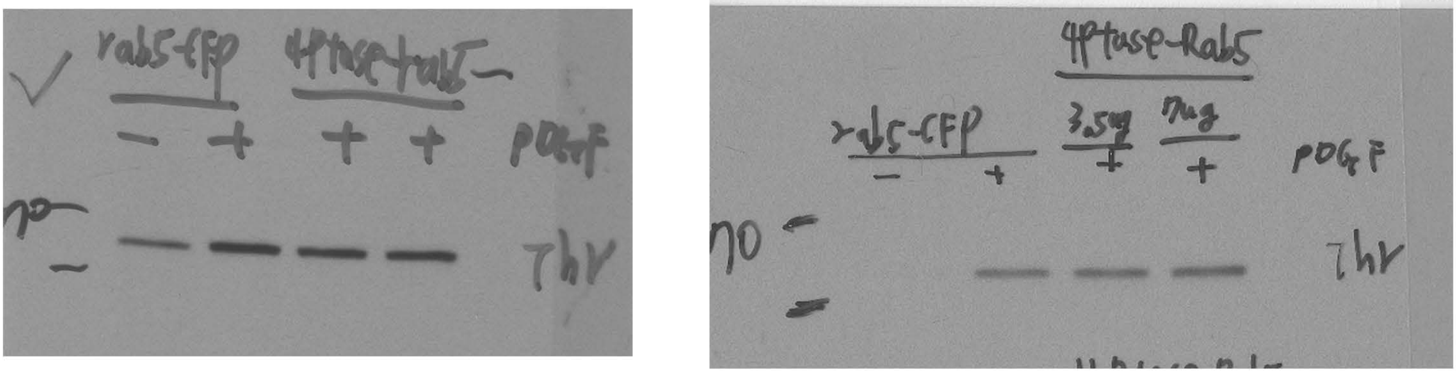

Fig 6A

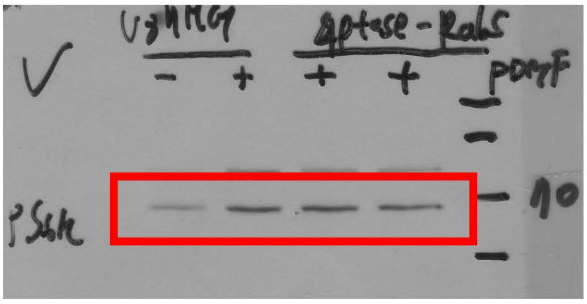

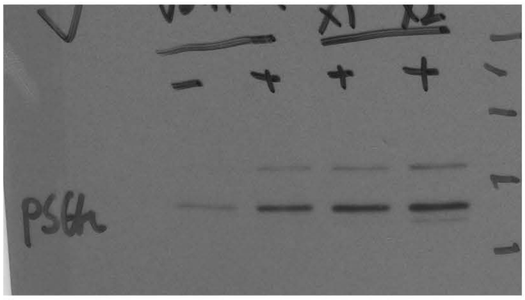

pS6K(Thr389)

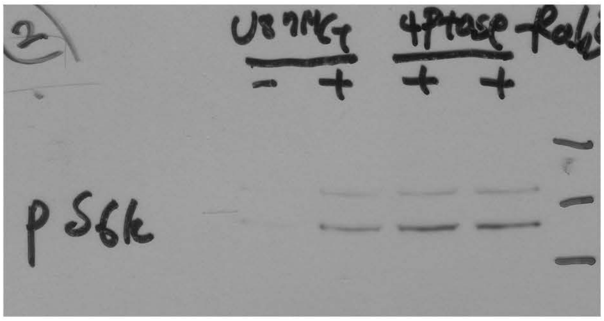

S6K

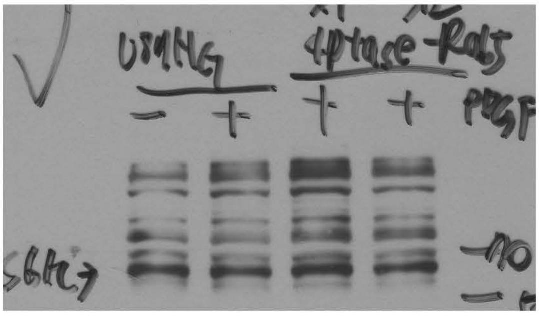

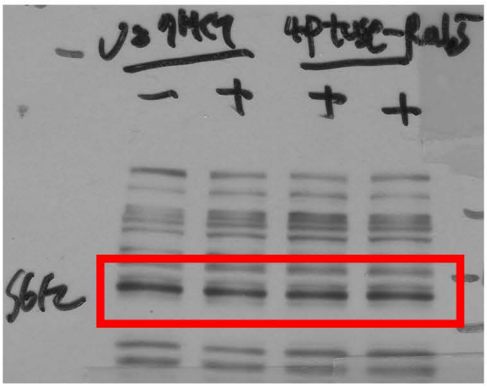

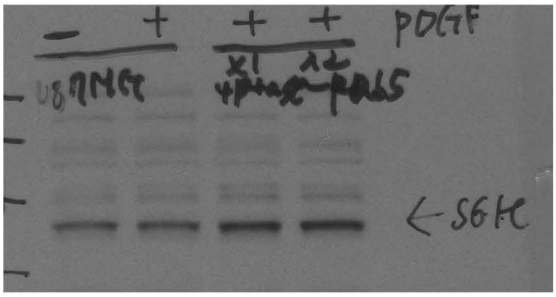

Rab5

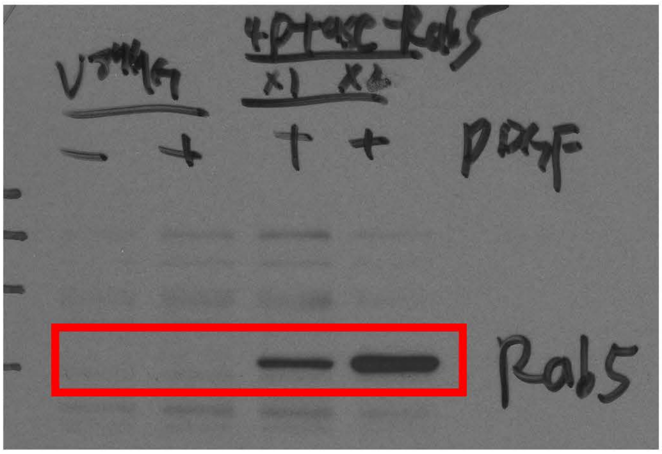

Fig 7A

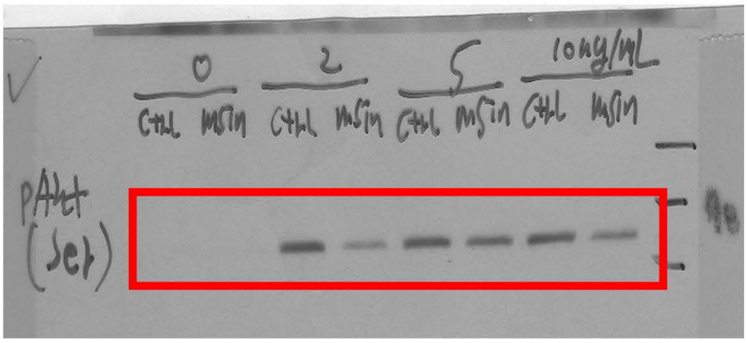

pAKT(Ser473)

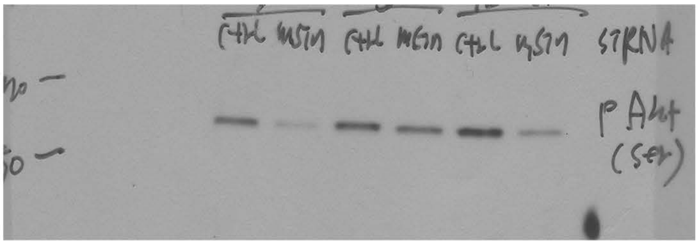

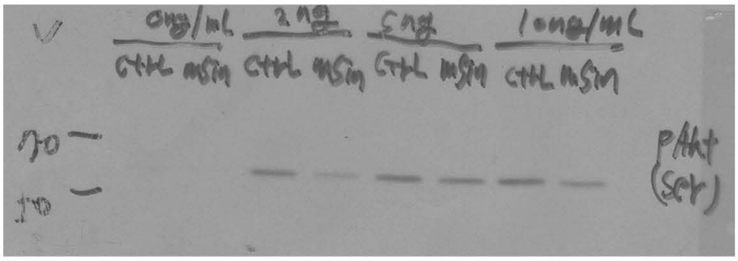

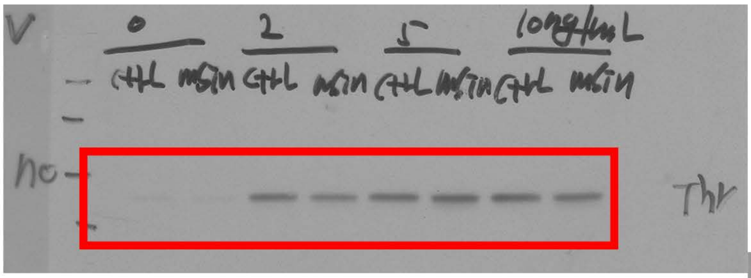

pAKT(Thr308)

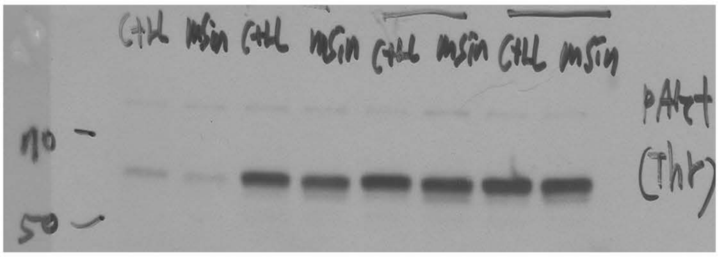

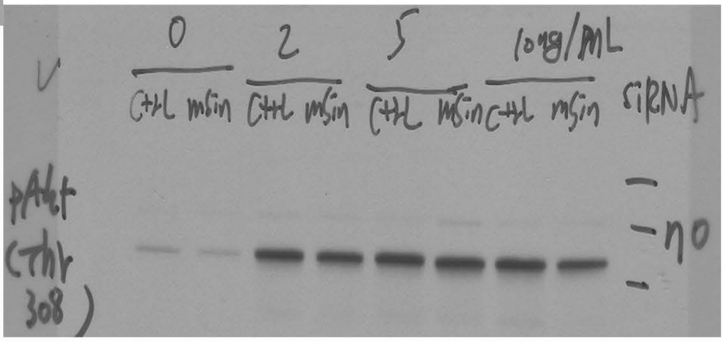

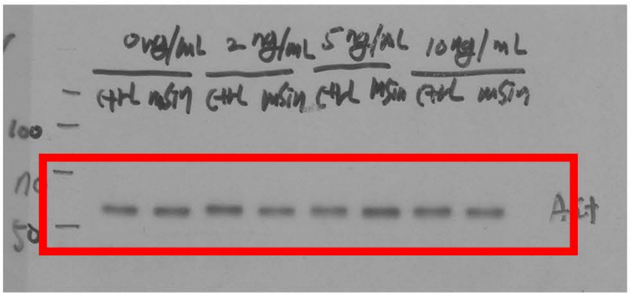

AKT

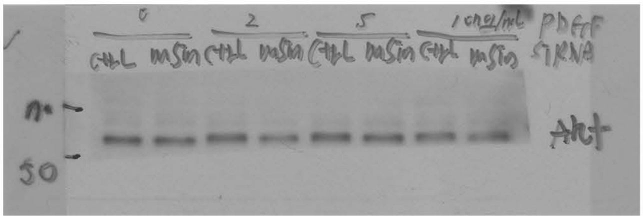

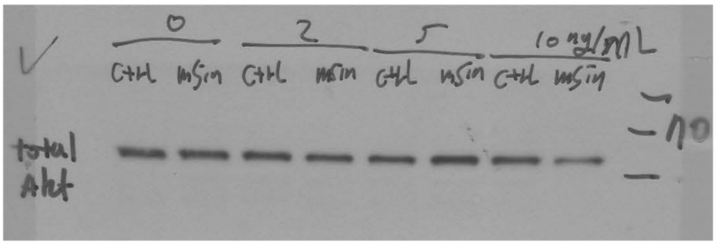

Fig 7A

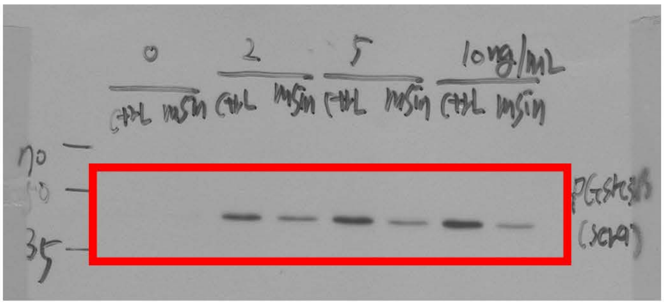
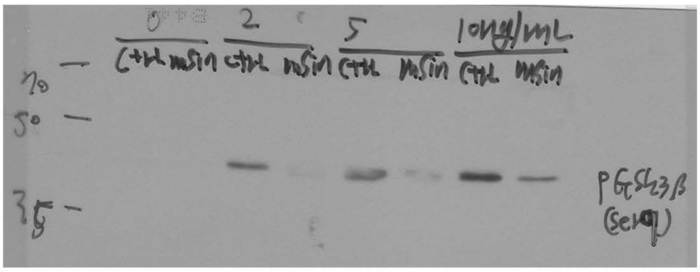

pGSK3 beta (Ser9)

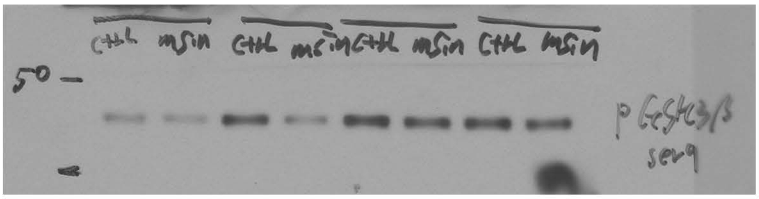

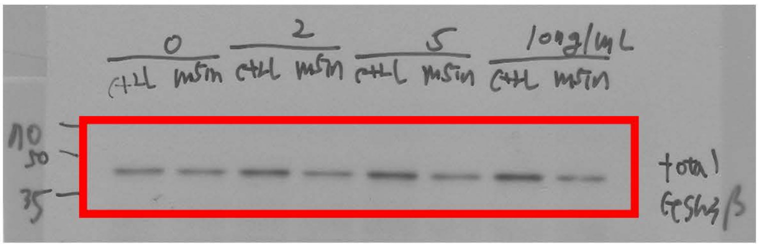

GSK3 beta

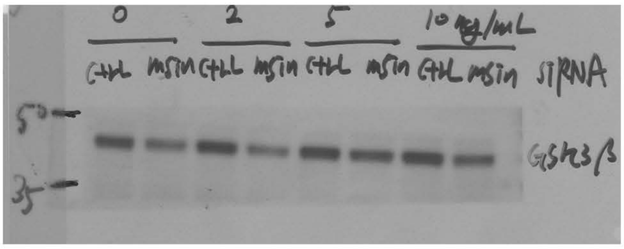
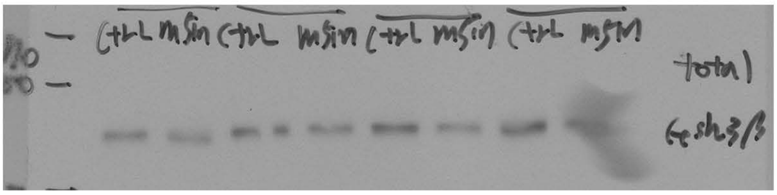

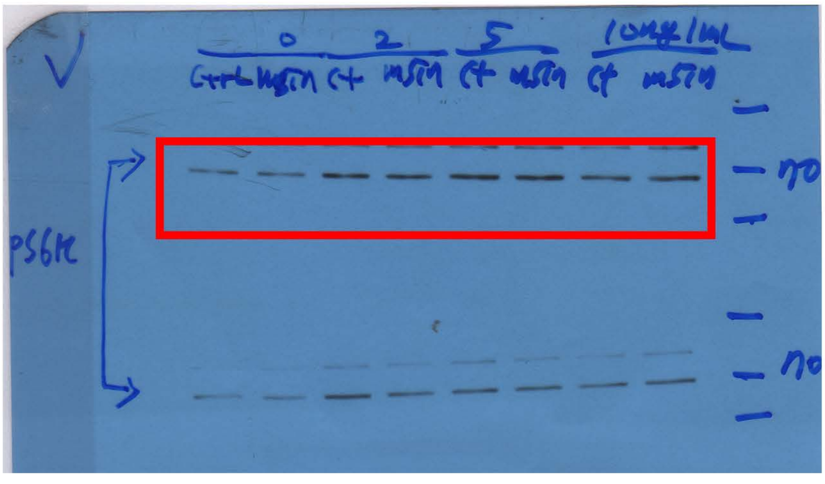

pS6K(Thr389)

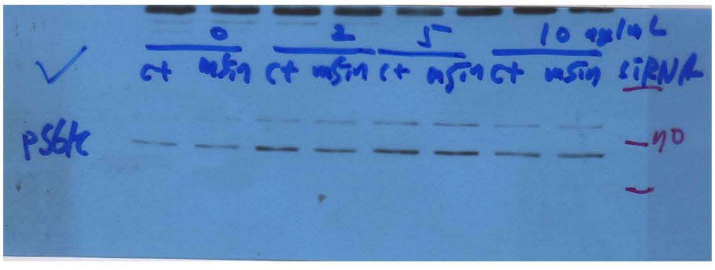

Fig 7A

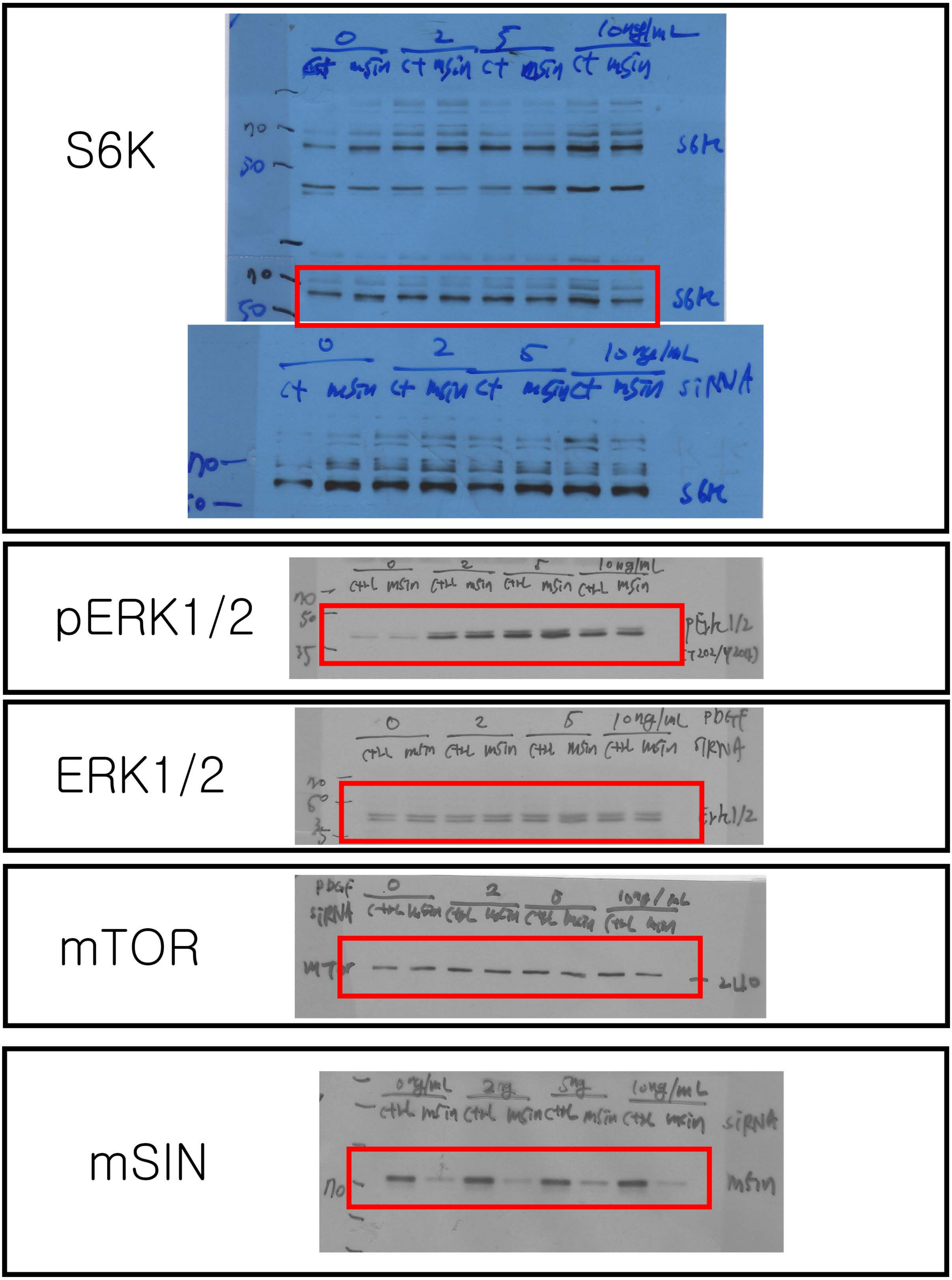

Supplement: Supplementary file 1 [file cancers-13-02405-s001.zip › Figure S9. full western blots.pdf]
