# Supplementary material for: Endosomal mTORC2 Is Required for Phosphoinositide-Dependent AKT Activation in Platelet-Derived Growth Factor-Stimulated Glioma Cells
Source: Cancers (Basel). 2021 May 16;13(10):2405. doi: 10.3390/cancers13102405 (PMC8157044; doi:10.3390/cancers13102405)
Supplement: Supplementary file 1 [file cancers-13-02405-s001.zip › Figures S1-8.pdf]

cancers

MDPI

Supplementary Materials

Endosomal mTORC2 Is Required for Phosphoinositide-Dependent AKT Activation in Platelet-Derived Growth Factor-Stimulated Glioma Cells

Suree Kim, Sukyeong Heo, Joseph Brzostowski and Dongmin Kang

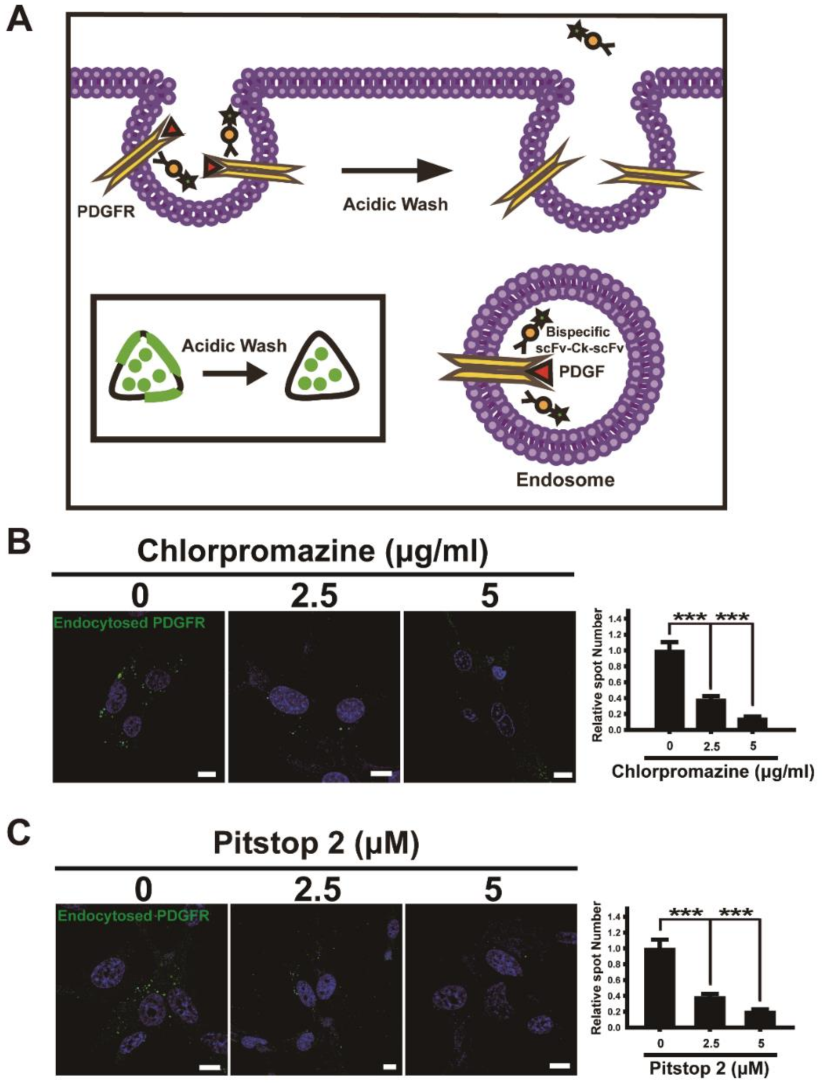

Figure S1. Chlorpromazine and Pitstop 2 inhibit PDGFR-mediated endocytosis. (A) Schematic representation of the endocytosis of bispecific anti-mPDGFRβ x cotinine scFv-Cκ-scFv fusion proteins and PDGF-BB. Washing with an acetic acid buffer (pH = 2.7) removes fluorescent antibodies bound to unendocytosed PDGFRs that remain at the surface of the plasma membrane. Fluorescence signals indicate endosomes, which result from receptor-mediated endocytosis. (B, C) Chlorpromazine and Pitstop 2 inhibit the endocytosis of bispecific anti-mPDGFRβ x cotinine scFv-Cκ-scFv fusion protein (green). U87MG cells were pretreated with chlorpromazine (2.5 μg/mL, 5 μg/mL) or Pitstop 2 (2.5 μM, 5 μM) for 3 min and then incubated with chlorpromazine or Pitstop 2 and bispecific anti-mPDGFRβ x cotinine scFv-Ck-scFv (10 μg/mL) for 5 min. Scale bar, 10 μm. The relative spot number was determined as the mean ± SEM from three independent experiments. The P-values were calculated using Student's two-tailed *t*-test. ***$P < 0.001$.

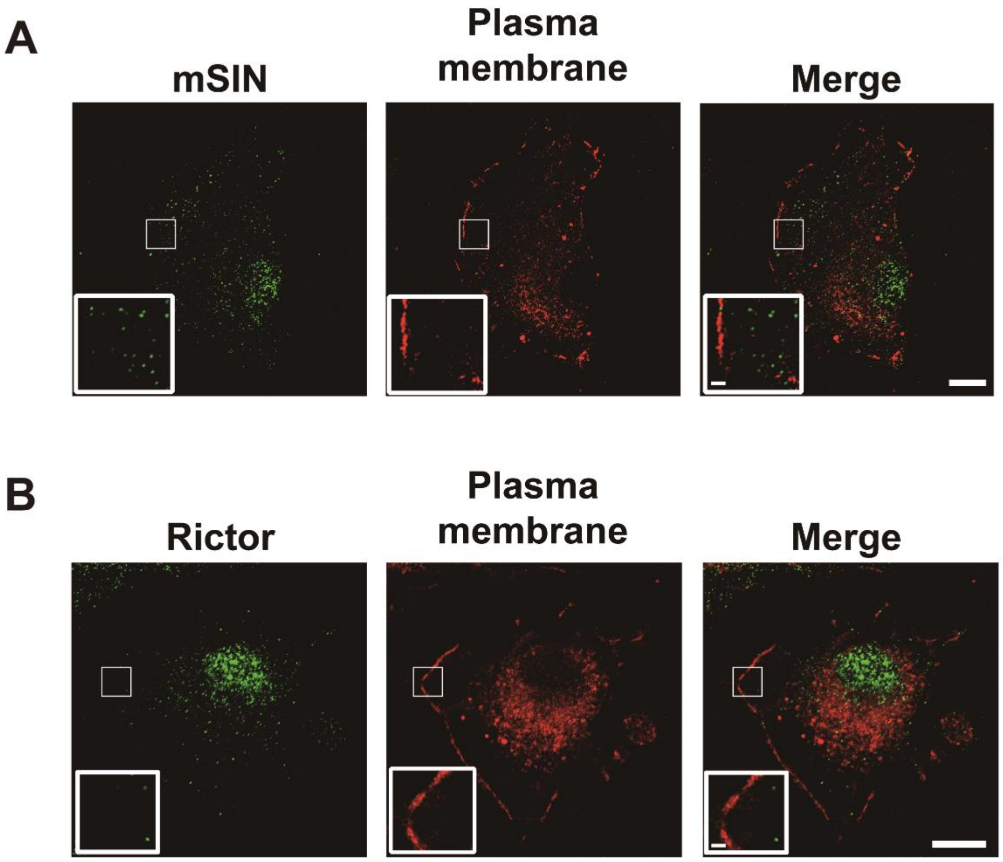

Figure S2. Most mSIN and Rictor, the two components of mTORC2, do not colocalize with the plasma membrane during growth factor activation. (A, B) Confocal microscopy of U87MG cells stained with CellMask plasma membrane dye (red) and antibodies to mSIN (*A*, green) and Rictor (*B*, green) during stimulation with PDGF-BB (50 ng/mL) for 5 min. The boxes are shown at higher magnification in the insets. Scale bar, 10 μm; insets, 1 μm.

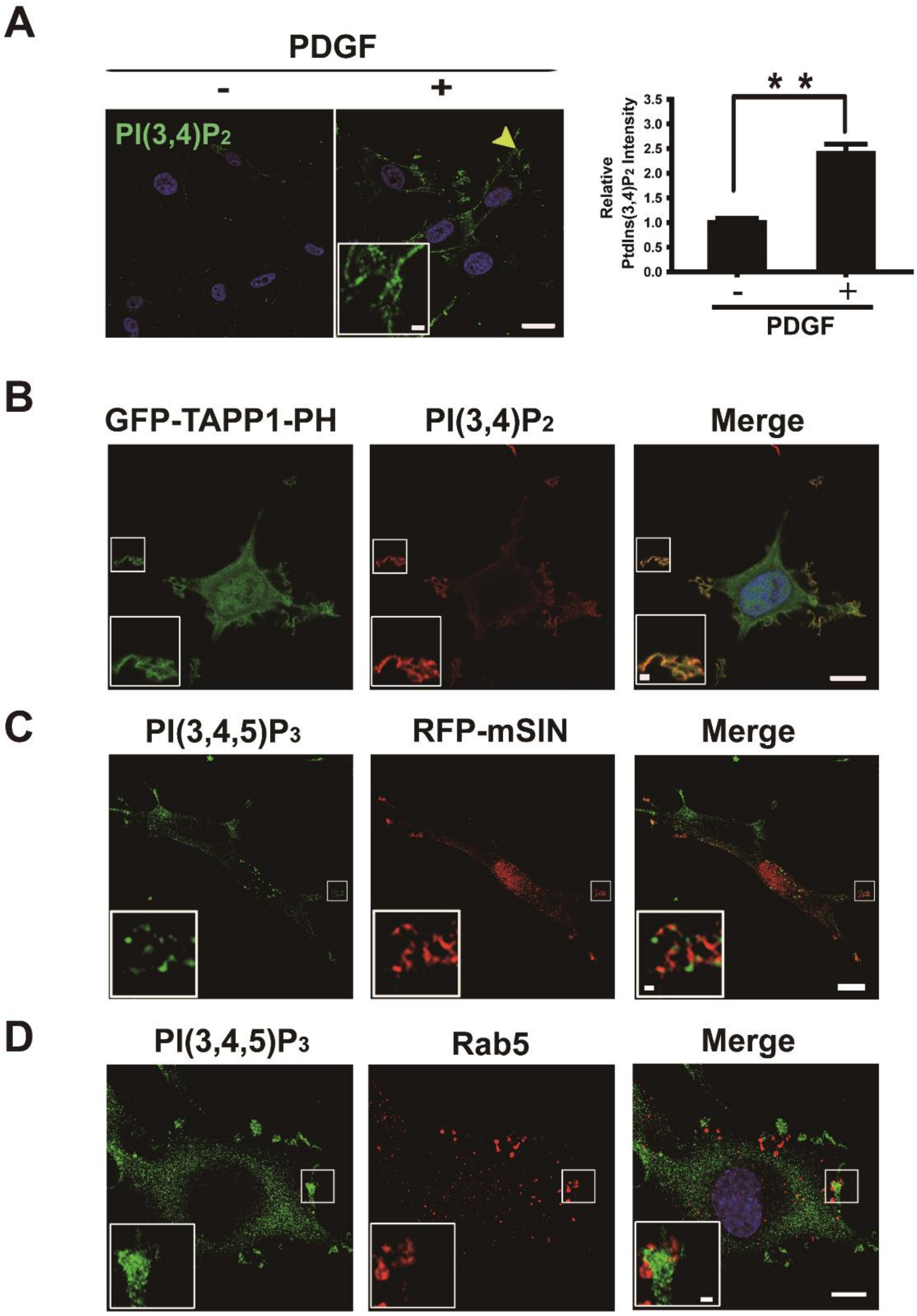

Figure S3. Accumulation of PtdIns(3,4,5)P_3 and PtdIns(3,4)P_2 in PDGF-stimulated cells. (A) Confocal imaging of U87MG cells activated with PDGF-BB (50 ng/mL) for 5 min and stained with an antibody to PtdIns(3,4)P_2 (green). Increased PtdIns(3,4)P_2 around the plasma membrane was observed in the presence of PDGF-BB. The area indicated by an arrowhead is shown at higher magnification in the inset. Scale bars, 20 µm; inset, 1 µm. The relative fluorescence intensity ratio of PtdIns(3,4)P_2 in the absence or presence of PDGF was determined as the mean ± SEM from three independent experiments (n = 9 cells), and the P-value was calculated using Student's two-tailed t-test. **P < 0.01. (B) Confocal microscopy of U87MG cells expressing GFP-TAPP1-PH (green), a reporter for PtdIns(3,4)P_2, and stained with an antibody specific to PtdIns(3,4)P_2 (red). The signals from the reporter and antibody overlapped. The white boxes are shown at higher magnification in the insets. Scale bars, 10 µm; insets, 1 µm. (C, D) Confocal microscopy of U87MG cells stained with an antibody to PtdIns(3,4,5)P_3 (green) and RFP-mSIN (red in C) or stained with an antibody to Rab5 (red in D). Representative images showed that PtdIns(3,4,5)P_3 rarely colocalized with mSIN and Rab5. The white boxes are shown at higher magnification in the insets. Scale bars, 10 µm; insets, 1 µm.

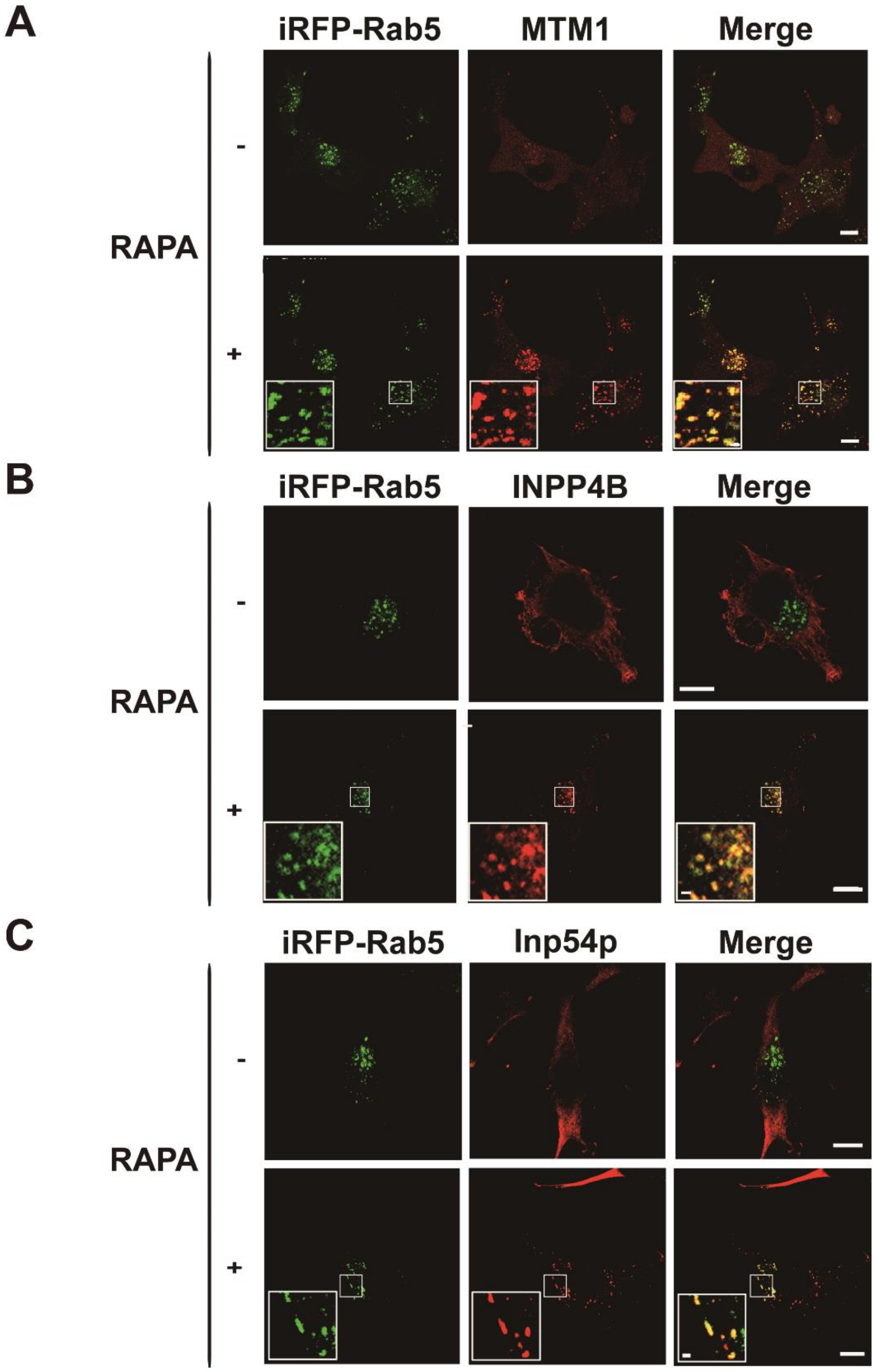

Figure S4. Heterodimerization of fluorescent FKBP-lipid phosphatases to endosome-anchored iRFP-FRB-Rab5 in response to a rapalogue (rapamycin, RAPA). (A-C) Confocal imaging of U87MG cells expressing iRFP-FRB-Rab5 (green) and mCherry-FKBP-MTM1 (A, red), mCherry-FKBP-INPP4B (B, red), or CFP-FKBP-Inp54p (C, red) in the presence of rapamycin (40 nM) for 5 min. Rapamycin treatment induced the recruitment of lipid phosphatase to early endosomes. The white boxes are shown at higher magnification in the insets. Scale bars, 10 μm; insets, 1 μm.

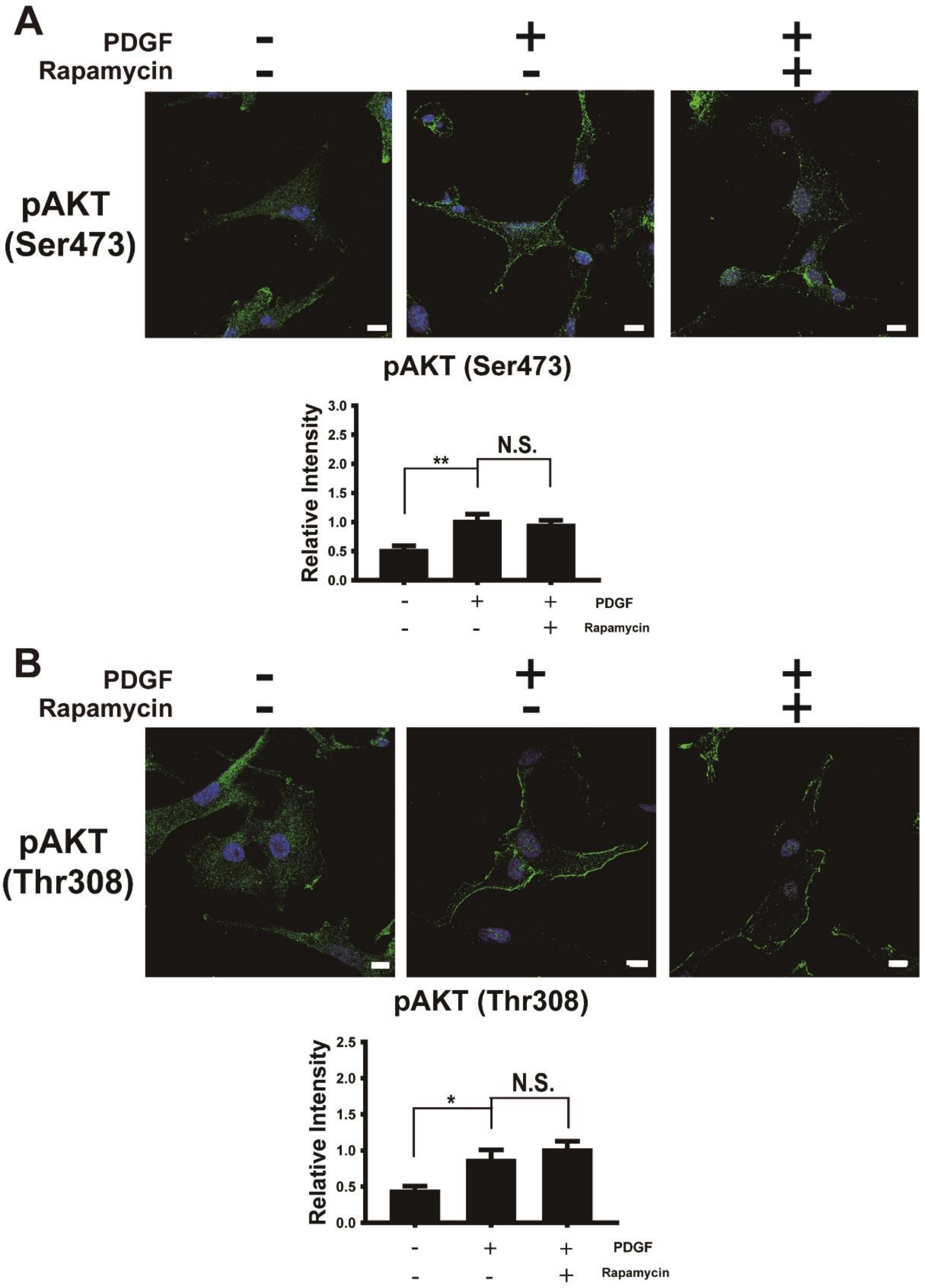

Figure S5. Phosphorylation of AKT at Ser473 and Thr308 remains constant during treatment with rapamycin of PDGF-stimulated U87MG cells. (A, B) Representative confocal images of U87MG cells upon rapamycin (40 nM) or PDGF-BB (50 ng/mL) activation for 5 min. Cells were fixed and stained with antibodies to pAKT(Ser473) (A, green) and pAKT(Thr308) (B, green). Scale bars, 10 μm. The relative intensity of pAKT(Ser473) and pAKT(Thr308) was evaluated and their level remained constant in the presence of rapamycin. Data are presented as the mean ± SEM from three independent experiments (n = 13 cells examined for each experiment), and P-values were calculated using Student's two-tailed t-test. *P < 0.05, **P < 0.01, N.S., not significant.

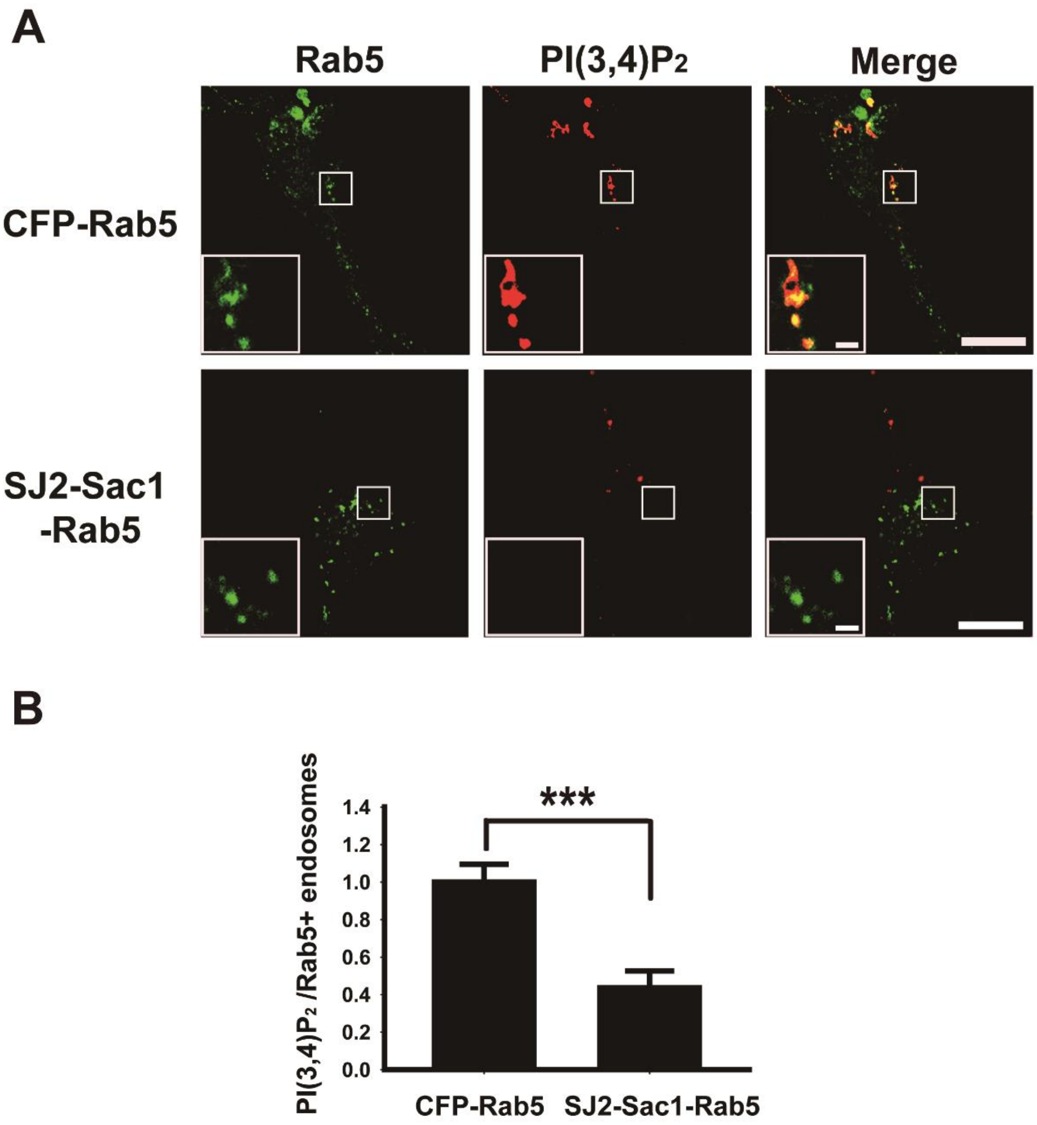

Figure S6. Expression of SJ2-Sac1-Rab5, an endosome-targeting 4-phosphatase, reduces the level of endosomal PtdIns(3,4)P_2. (A) Confocal imaging of U87MG cells expressing CFP-Rab5 or SJ2-Sac1-Rab5 and stained with antibodies to Rab5 (green) and PtdIns(3,4)P_2 (red). The white boxes are shown at higher magnification in the insets. Scale bars, 10 µm; insets, 1 µm. (B) The relative fluorescence intensity ratio of PtdIns(3,4)P_2 to CFP-Rab5 or SJ2-Sac1-Rab5 was also evaluated at early endosomes. Data are presented as mean ± SEM from three independent experiments, and the P-value was calculated using Student's two-tailed *t*-test. ***$P < 0.001$.

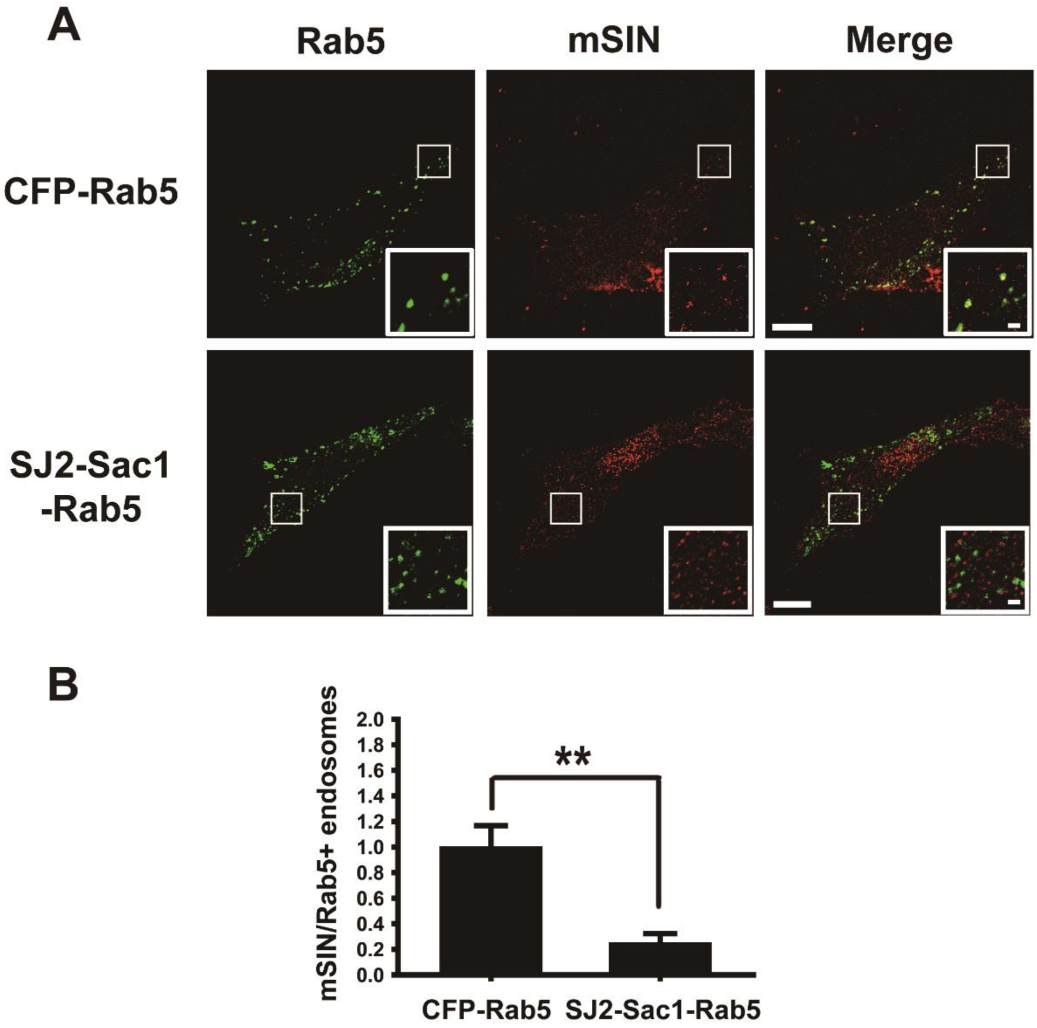

Figure S7. Expression of SJ2-Sac1-Rab5, an endosome-targeting 4-phosphatase, reduces the level of endosomal mSIN, an mTORC2 component. (A) Confocal imaging of U87MG cells expressing CFP-Rab5 or SJ2-Sac1-Rab5 and stained with antibodies to Rab5 (green) and mSIN (red). The areas indicated by white boxes are shown at higher magnification in the insets. Scale bars, 10 µm; insets, 1 µm. (B) The relative fluorescence intensity ratio of mSIN to CFP-Rab5 or SJ2-Sac1-Rab5 was also evaluated at early endosomes. Data are presented as mean ± SEM from three independent experiments, and the P-value was calculated using Student's two-tailed t-test. **$P < 0.01$.

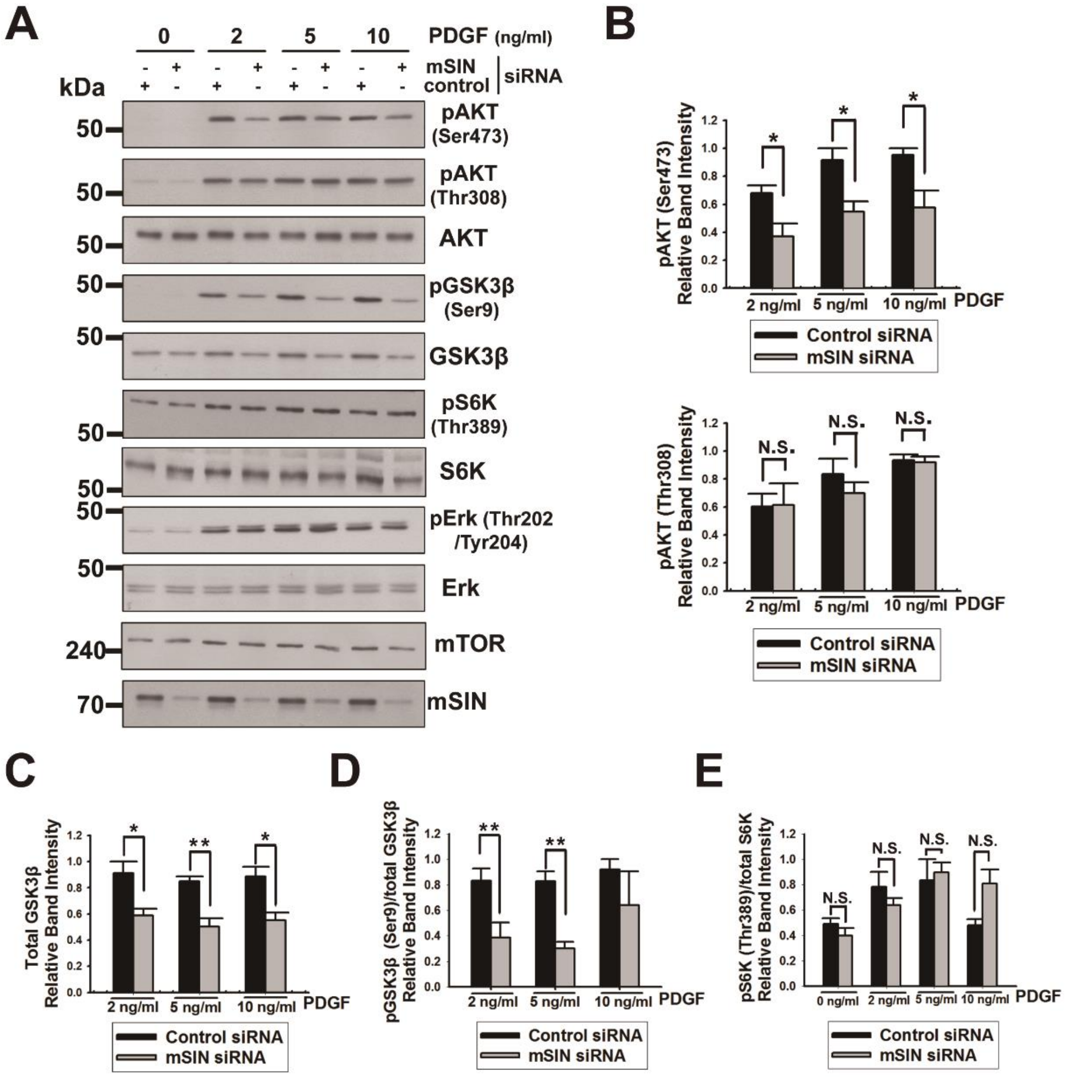

Figure S8. AKT signaling via Ser473 phosphorylation is regulated by mTORC2 in PDGF-stimulated cells. (**A**) U87MG cells were transiently transfected for 48 h with control siRNA or siRNA against mSIN, serum starved for 5 h, and incubated for 3 min in the presence of the indicated concentrations of PDGF-BB. Cell lysates were subjected to immunoblot analysis with antibodies to the indicated proteins. (**B-E**) The relative immunoblot signal intensity of pAKT(Ser473) (**B**), pAKT(Thr308) (**B**), GSK3β (**C**), pGSK3β(Ser9) normalized by that of GSK3β (**D**), and pS6K(Thr389) (**E**) were determined as the mean ± SEM from three independent experiments, and P-values were calculated using Student's two-tailed *t*-test. *P < 0.05, **P < 0.01, N.S., not significant.

Our work revealed the reduction of mTORC2 activity by mSIN depletion and its effect on AKT activity and the activation of its substrate in U87MG cells. A reduction in the levels of pAKT(Ser473), pGSK3β(Ser9), and total GSK3β was observed in mSIN knockdown cells compared to siCont-treated cells, whereas the levels of pAKT(Thr308), pS6K(Thr389), and pErk(Thr202/Tyr204) remained unchanged between simSIN and siCont cells (Figure S8). This result indicates that in PDGF-activated U87MG cells, reduced mTORC2 activity by downregulating mSIN1 expression specifically reduces AKT phosphorylation at Ser473 and downstream substrates, including GSK3β, without affecting the phosphorylation of pS6K, an mTORC1 substrate. These findings are similar to previous works using mSIN knockout mouse [43] and mSIN knockdown cells [72].

Legends for Movies

Video S1 (separate file). Confocal images of live U87MG cells expressing GFP-mSIN-RBD-PH activated with PDGF-BB (50 ng/mL) at 30-s intervals for 15 min. Arrows indicate GFP-mSIN-RBD-PH puncta.

Video S2 (separate file). Confocal images of live U87MG cells expressing GFP-mSIN-RBD-PH and mCherry-AKT-PH (a reporter for PtdIns(3,4,5)P_3 and PtdIns(3,4)P_2) activated with PDGF-BB (50 ng/mL) at 30-s intervals for 15 min. Arrows indicate yellow puncta showing mSIN-RBD-PH (green) colocalized with AKT-PH (red).

Video S3 (separate file). Confocal images of live U87MG cells expressing iRFP-FRB-Rab5 (green) and mCherry-FKBP-MTM1 (red) treated with rapamycin (40 nM) at 30-s intervals for 10 min.
